# Intraoperative injection of absorbable gelatin sponge (AGS) mixed with cement followed by spinal decompression to treat elderly with vertebral hemangiomas

**DOI:** 10.1186/s12891-020-3143-6

**Published:** 2020-02-24

**Authors:** Weihong Xu, Zhibin Lan, Yuming Huang

**Affiliations:** 1 Department of Spine Surgery, First Affiliated Hospital of Fujian Medical University, Fuzhou, 350004 Fujian China; 2 Department of Spine Surgery, Quanzhou Orthopedic-Traumatological Hospital of Fujian Traditional Chinese Medicine University, Quanzhou, 362000 Fujian China; 3 The Orthopedics Department, Fuzhou Second Hospital affiliated to Xiamen University, Cangshan District, Fuzhou, 350007 Fujian China

**Keywords:** Aggressive vertebral hemangiomas, Pain, Multimodal surgery, Absorbable gelatin sponge

## Abstract

**Background:**

Elderly patients with vertebral hemangiomas are rare and might require surgery. Thus, the choice of surgery for these lesions remains controversial because of the rarity of these lesions. This study aimed to analyze the clinical efficacy of the intraoperative injection of absorbable gelatin sponge mixed with cement followed by spinal decompression to treat the elderly with typical vertebral hemangiomas. The risk factors for hemangioma recurrence were investigated through a literature review.

**Methods:**

We retrospectively analyzed 13 patients with typical aggressive hemangiomas between January 2009 and January 2016. Of these patients, 7 were treated with spinal decompression combined with intraoperative vertebroplasty (Group A), and 6 patients were treated with decompression with intraoperative vertebroplasty and absorbable gelatin sponge (Group B). The general data and perioperative data of the patients were compared. Patients were followed up for at least 3 years, and postoperative complications and recurrence rates were recorded and compared.

**Results:**

All patients had typical aggressive hemangiomas. The average age of all patients was 64.4 ± 3.3 years. The preoperative data did not differ significantly between the two groups (*P* > 0.05). The blood loss of groups A and B was 707.1 ± 109.7 ml and 416.7 ± 103.3 ml, respectively (*P* = 0.003) (P = 0.003), and the average surgery durations were 222 ± 47.8 min and 162 ± 30.2 min, respectively (*P* = 0.022). The average follow-up duration was 62 ± 19 months, and no cases of recurrence were found at the final follow-up assessment.

**Conclusions:**

Multimodal treatment significantly alleviated the clinical symptoms of elderly patients with typical aggressive vertebral hemangiomas. Intraoperative absorbable gelatin sponge injection is a safe and effective way to reduce blood loss and surgery duration.

## Background

A vertebral hemangioma is a benign tumor. Anatomical and imaging studies have shown that the incidence of vertebral hemangiomas ranges from 10 to 26%; most cases are asymptomatic, and only 0.9–1.2% of affected individuals have clinical symptoms [1, 2]. Pathologically, a hemangioma is composed of benign vascular dysplasia or vascular lumen and endothelial cells. These lesions are usually localized to one of three anatomical locations: on the periosteal surface, within the cortex, or within the medullary canal [3]. On imaging, the spinal vertebral body typically manifests as fence-, grid-, and honeycomb-like changes to the vertebral body [4, 5]. Currently, a vertebral hemangioma is classified using the following three stages of the Enneking system: (1) during latency, the tumor is restricted within the spinal compartment (Enneking stage 1; S1); (2) during the active stage, the tumor is contained within the spinal compartment and is accompanied by clinical symptoms (Enneking stage 2; S2); and (3) during the aggressive stage, the tumor expands out of the spinal compartment and is accompanied by clinical symptoms (Enneking stage 3; S3) [1].

Because S3 stage hemangiomas are rare with a high recurrence rate, simple surgical approaches remain controversial. Some experts [6, 7] recommended simple spinal decompression or tumor reductive surgery, which has low technical surgery requirements, causes little intraoperative blood loss, and is associated with fast postoperative recovery. However, the high recurrence rate leads to poor clinical prognosis [2, 8–11]. Therefore, some authors have proposed en bloc resection for vertebral hemangiomas [3, 12]. According to the currently available data, no tumor recurrence has been found after en bloc resection. However, the intraoperative blood loss is far more than that of decompression, and the incidence of bleeding-related complications after surgery is significantly increased. Furthermore, this procedure not only demands surgical skill but also requires patients with higher health statuses [12]. Thus, some experts have recently studied multimodal treatments for aggressive hemangiomas [13–16]. Multimodal treatments include preoperative interventional embolization, spinal canal decompression or en bloc.

Vertebral hemangiomas are common among people 40–50 years of age but are relatively rare among elderly patients (> 60 years old). To the best of our knowledge, no study has analyzed the treatment of elderly patients with typical S3 hemangiomas. Therefore, we evaluated the clinical efficacy and long-term recurrence rate of the multimodal treatment of elderly patients with S3 hemangiomas and investigated whether the intraoperative injection of absorbable gelatin sponge affected bleeding or relevant complications.

## Methods

After approval by the ethics committee, we retrospectively analyzed the cases of elderly patients with typical S3 hemangiomas (> 60 years old) undergoing surgery at our hospital between January 2009 and January 2016. We consecutively recruited 13 patients and tracked their progression for at least 3 years. Seven patients were treated with spinal decompression combined with intraoperative vertebroplasty (Group A), and six patients were treated with absorbable gelatin sponge combined with decompression and vertebroplasty (Group B) since 2013. All procedures were performed by one spinal surgeon.

Experienced spinal surgeons collected general patient information (including sex, age, medical history, tumor level, fusion level, body mass index (BMI), main clinical symptoms, and symptom duration). The Frankel grade classification was used to assess neurologic status. Back pain was assessed using a visual analogue scale (VAS). The perioperative data included surgical procedure, intraoperative blood loss, surgery duration, postoperative hospital stay duration, presence of complications, and postoperative pathology.

### Imaging and biopsy

**Fig. 1:**
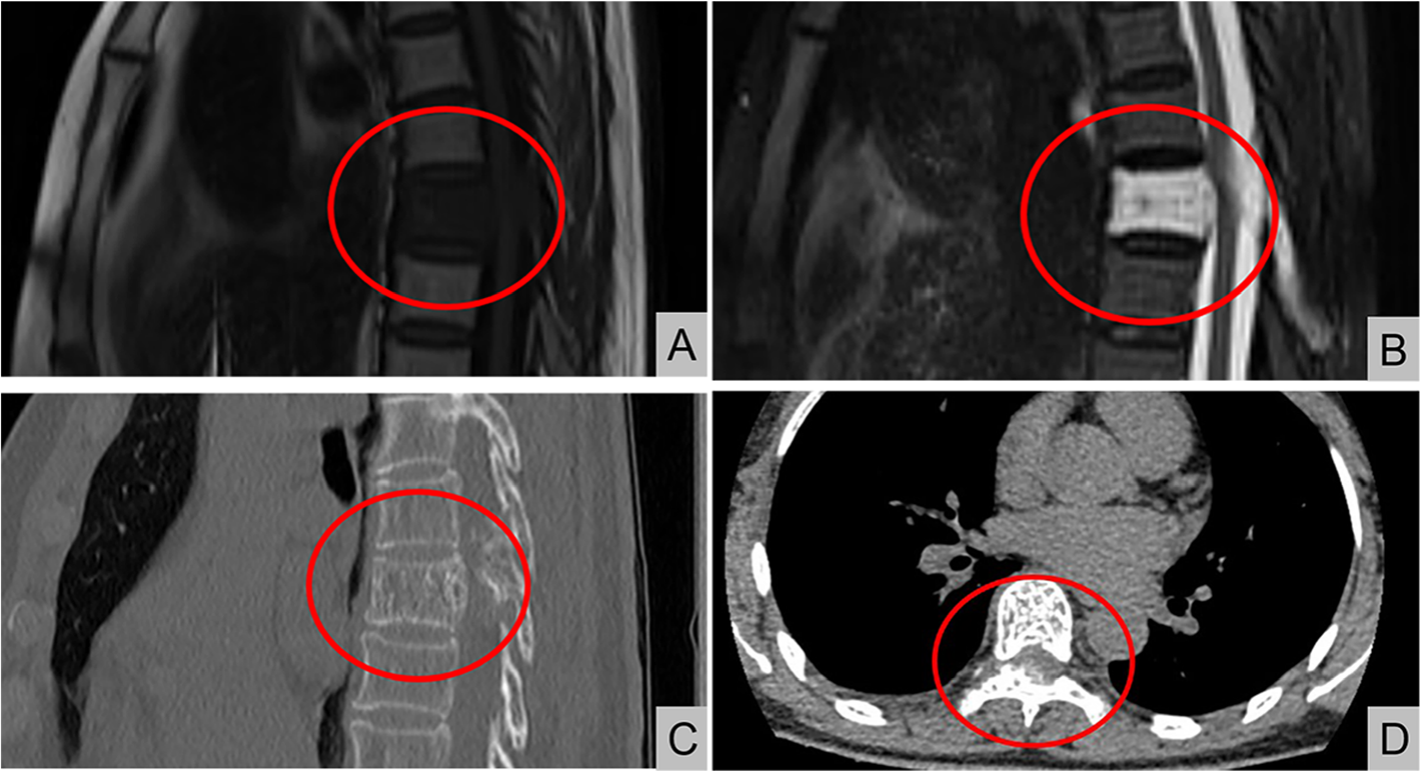
A 69 year-old female patient who had a T7 hemangiomas (typical MRI and CT scans)

All patients received preoperative lateral standing radiographs, computed tomography (CT) scans, and magnetic resonance imaging (MRI) of the spine. Typical imaging manifestations included “honeycomb” and “corduroy” signs on CT and thickened trabecular bone on the cross-section in the form of a dot. MRI examinations showed low T1 signal/high T2 signal changes in the affected spine (Fig. 1). Lateral standing radiographs and MRI imaging of the spine were taken immediately after surgery. Lateral radiographs of the spine were taken 3 months after surgery and during the follow-up period to confirm and determine the status of the implants.

### Surgical decisions

Surgery was usually performed for patients with typical S3 hemangiomas to alleviate their clinical symptoms. Surgical decompression was the primary option for elderly patients, regardless of whether the tumor invaded the surrounding soft tissue. The spine was stabilized with 4 or 8 pedicle screws depending on the bone condition (8 pedicle screws for severe osteoporosis patients). In this study, we performed preoperative embolization for all patients undergoing S3 hemangioma surgery to reduce intraoperative blood loss. We injected cement through the unilateral pedicles of the vertebral arch for all patients (a unilateral pedicle approach was first considered). Postoperative radiotherapy was not performed for elderly patients.

### Surgical methods

**Fig. 2:**
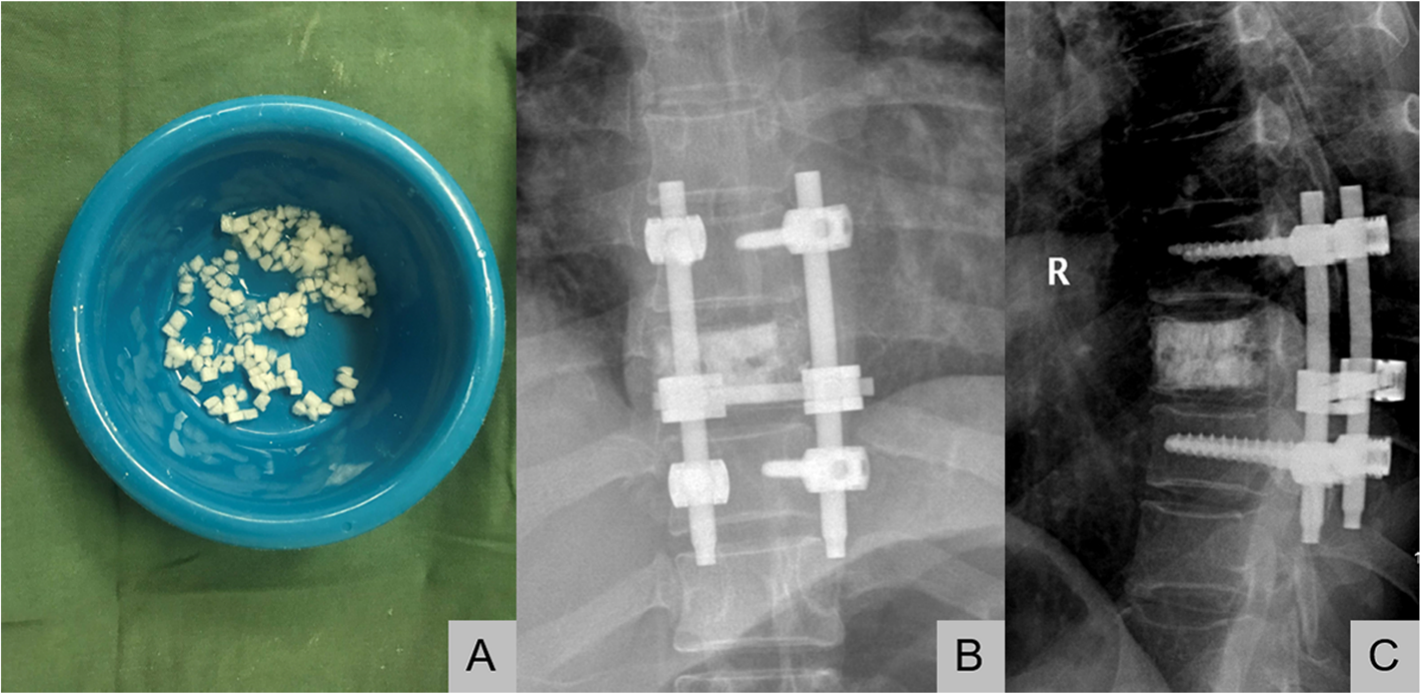
Photograph showing pre-prepared 1 mm × 1 mm × 1 mm AGS particles mixed with the contrast agent and C-arm fluoroscopy photograph of a 69-year-old woman who underwent decompression and intraoperative vertebroplasty with absorbable gelatin sponge

A posterior median incision centered on the affected spinous process was made to expose 1–2 vertebral joints above and below the vertebral plates. Then, the pedicles of the vertebral arch were punctured under direct visualization guided by C-arm fluoroscopy. Pre-prepared 1.0 mm × 1.0 mm × 1.0 mm absorbable gelatin sponge (Jinling Pharmaceutical Co., Ltd., Nanjing, China) particles were mixed with the contrast agent and injected into the affected spines of patients in group B, and 6~8 ml viscous cement was used to fill the affected spine under visualization guided by C-arm fluoroscopy. The puncture needle was withdrawn after the cement solidified. Patients in group A were only injected with cement, and the remaining procedure was the same. The lesion and entire vertebral body needed to be packed without bone cement leakage to destroy and shrink the malformation. Because bone cement can release energy during expansion, the local temperature reaches 80–90 degrees Celsius, and the diseased site can shrink. Then, direct decompression of the lesion (laminectomy) was performed to remove the invasive hemangiomas. (Fig. 2).

### Statistical analyses

All data were analyzed using SPSS 23.0 (SPSS Inc., Chicago, IL). The Kolmogorov-Smirnov test was performed to determine if the data were normally distributed. Normally distributed data are presented as the means±standard deviations. Nonnormally distributed data are represented as medians and ranges. Significant differences in data between groups were determined via independent sample U-tests and t-tests. *P* < 0.05 was considered significant.

## Results

**Fig. 3:**
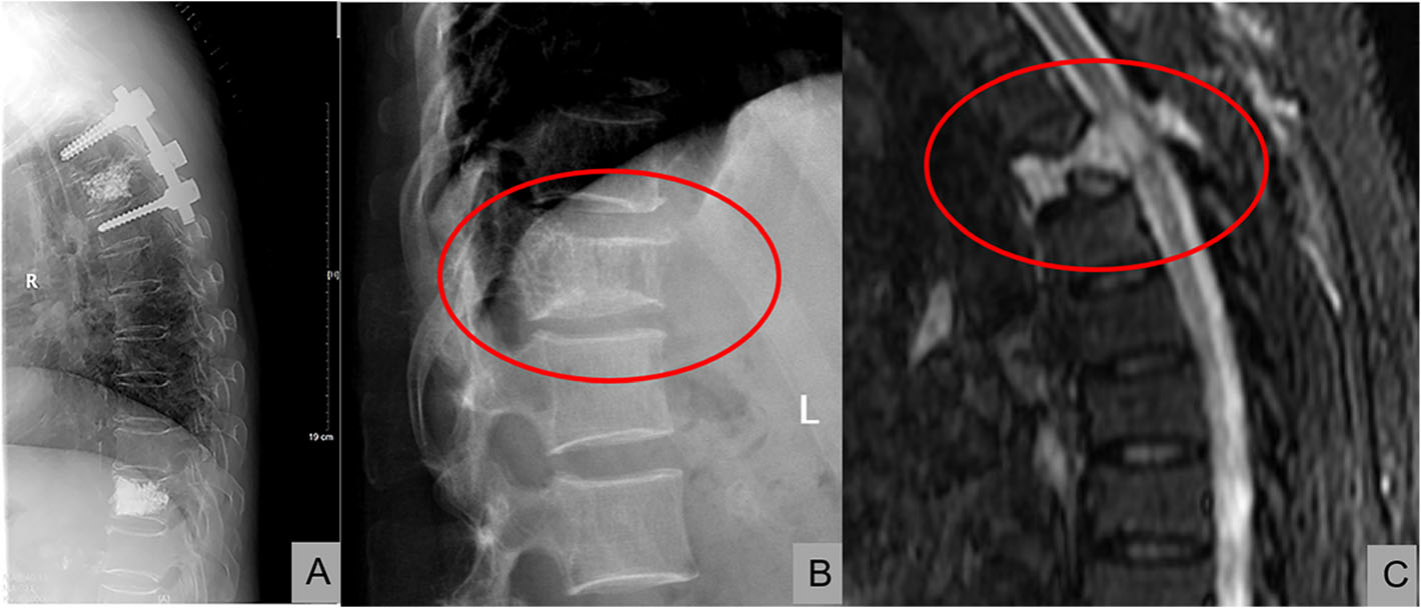
**a** shows a 62-year-old male patient who had multiple hemangiomas (T2, T10), and the hemangioma at T2 underwent decompression and intraoperative vertebroplasty. The T10 tumor only underwent vertebroplasty. **b** and **c** show a 62-year-old female patient who had pathological fractures at T4

A total of 13 elderly patients (four men and nine women) were recruited for this study, with an average age of 64.4 ± 3.3 years. Twelve cases of aggressive hemangioma were in the thoracic vertebra, and one was in the lumbar vertebra. One patient had multiple hemangiomas (T2, T10), and only the patient with a hemangioma at T2 underwent decompression treatment. The T10 tumor was asymptomatic and was only subjected to percutaneous vertebroplasty. Ten patients reported back pain with a VAS score of 6 points (6–8 points). Five patients had myelopathic symptoms (four patients had Frankel grade D, and one patient had Frankel grade C). Two patients had radicular symptoms, and seven patients had pathological fractures. Two cases involved the bilateral pedicles, and 11 only involved the unilateral pedicles. Twelve cases involved the vertebral canal, and the tumors of three patients spread to the surrounding soft tissue. Two patients who underwent preoperative CT biopsy confirmed that they had hemangiomas. The preoperative data of the two groups of patients did not differ significantly (*P* < 0.005) (Fig. 3).

The general and perioperative data are shown in Tables 1 and 2.

**Table 1:** General data

No.	Age(y)	CDH	tumor Level	Fusion Levels	symptoms	BMI	Symptom duration (mo)
1	63	–	T8	T7–9	Pain	19.5	5
2	66	HBP	T3	T1–5	Pain, myelopathy	20.0	1
3	62	–	T10, T2	T9–11	Pain, myelopathy	22.0	12
4	61	HBP	L4	L3–5	Radiculomyelopathy	24.2	36
5	68	DM	T12	T11-L1	Radiculomyelopathy	23.3	3
6	71	–	T6	T5–7	Pain	20.0	24
7	65	–	T4	T3–5	Pain, myelopathy	19.9	6
8	69	–	T7	T5–9	Pain	17.5	30
9	65	HBP	T12	T11-L1	Radiculomyelopathy	19.9	12
10	62	–	T7	T6–8	Pain, myelopathy	20.2	3
11	61	–	T3	T2–4	Pain	25.0	9
12	62	DM	T4	T3–5	Pain, myelopathy	24.0	6
13	62	–	T3	T2–4	Pain	21.7	9

*Abbreviations*: *CDH* chronical diease history, *HBP* high blood pressure, *DM* diabetes mellitus, *BMI* body mass index

**Table 2:** Perioperative data

No.	TA	quantity of VP (ml)	Group	blood loss (ml)	surgery time (min)	Time to discharge after surgery (d)	blood transfusion	Pre-Frankel	Pre-VAS	Complication
1	Y	6	Group A	700.0	120	7		–	9	–
2	Y	6	Group A	800.0	240	8	RBC 4u	D	8	–
3	Y	6	Group A	650.0	140	7		D	8	–
4	Y	bilateral 3	Group A	900.0	300	10	RBC 4u	–	–	–
5	Y	7	Group A	600.0	240	7		–	–	–
6	Y	6	Group A	700.0	200	10		–	7	–
7	Y	8	Group A	600.0	240	7		D	6	–
8	Y	6	Group B	550.0	200	7	FFP 200 ml	C	6	–
9	Y	7	Group B	300.0	130	7		–	–	–
10	Y	6	Group B	450.0	150	10		D	7	–
11	Y	7	Group B	500.0	150	7		–	6	–
12	Y	6	Group B	400.0	120	9		D	6	–
13	Y	7	Group B	300.0	160	7		–	6	–

*Abbreviations*: *Group A* decompression combined with intraoperative vertebroplasty, *Group B* decompression combined with intraoperative vertebroplasty and absorbable gelatin sponge, *TA* transarterial embolization, *Y* yes, *RBC* red blood cell, *FFP* fresh freezing plasma, *VAS* Visual analogy score, *Pre-* preoperative

### Surgical comparisons

The average intraoperative blood loss and surgery duration differed significantly between the two groups (*P* = 0.003 and 0.022, respectively; Table 3).

**Table 3:** Comparisons between operations

Factors	Group A	Group B	t	P
Blood loss (ml)	707.1 ± 109.7	416.7 ± 103.3	4.889	0.003
Surgery time (min)	222 ± 47.8	162 ± 30.2	2.658	0.022
Discharge (d)	15 ± 1.5	17.0 ± 3.4	0.219	0.831

*Abbreviations*: *Group A* decompression combined with intraoperative vertebroplasty, *Group B* decompression combined with intraoperative vertebroplasty and absorbable gelatin sponge, *VP* vertebroplasty, *AGS* absorbable gelatin sponge;

Because of the large amount of intraoperative blood loss, two patients in group A were infused with 4 U of suspended red blood cells during surgery. One patient in group B had a large amount of postoperative drainage due to abnormal blood coagulation and was infused with 200 ml of fresh frozen plasma to improve coagulation. The drain was removed within 2–4 days. No complications occurred. Postoperative pathology tests confirmed that all patients had hemangiomas.

### Follow-up period

All patients were followed up clinically for an average of 62 ± 19 months. Because intraoperative injections of absorbable gelatin sponge were implemented in 2013, the average follow-up duration of group A patients was 76 ± 12 months, and the average follow-up duration of group B patients was 46 ± 7 months. No myelopathic or radiculopathic symptoms were observed at the follow-up assessment (Frankel grade E). The VAS score was 1 point (0–2 pints), which was significantly lower than that prior to the surgery (*P* < 0.05). No patients had tumor recurrence at the last follow-up assessment.

## Discussion

Typical S3 hemangiomas can cause spinal cord compression, bone destruction, and neurological damage. Because of the small number of cases, no consensus exists concerning the gold standard to treat S3 hemangiomas. Currently, the reported treatments for vertebral hemangiomas include radiotherapy [17], interventional embolization [18], alcohol ablation [19, 20], vertebroplasty [21], and surgery [2, 3, 8]. Cloran et al. [4] believed that physicians should attend to symptomatic hemangiomas and that multimodal treatments should be used for patients with S3 hemangiomas, which includes preoperative interventional embolization, spinal canal decompression or en bloc. We started thinking about an absorbable gelatin sponge to reduce the amount of bleeding from 2013. Since there was less bleeding, we maintained a clear field of vision and enabled complete removal of the abnormal tissue during the procedure. This was the first study to apply multimodal treatments for elderly patients with aggressive hemangiomas to investigate their postoperative efficacy and intraoperative complications and reported the clinical efficacy of absorbable gelatin sponge infusion during spinal decompression surgery.

In this study, we performed preoperative embolization for all patients undergoing S3 hemangioma surgery to reduce intraoperative blood loss. Robinson et al. [22] compared the intraoperative blood loss of patients who underwent preoperative embolization with that of those who did not undergo preoperative embolization and found that preoperative embolization significantly reduced intraoperative blood loss. Other additional factors, including anesthesia, surgical skill, and patient coagulation, might also cause intraoperative blood loss. As early as 1972, Hekster et al. [18] reported that preoperative embolization effectively reduced intraoperative blood loss and blood loss-related complications. In this study, all elderly patients underwent preoperative embolization; after surgery, no patients had blood loss-related complications. These findings are consistent with the literature reviewed above.

Acora et al. [3] believed that en bloc resection was required for patients with tumors that spread outside of the vertebral body, which significantly reduced tumor recurrence. The main advantage of en bloc resection was the low recurrence rate; however, some experts selected this method with hesitation because of the large amount of intraoperative blood loss, high technical requirements, and multiple complications. The study of Tomita et al. [12] and Ogawa et al. [23] revealed that the treatment of aggressive hemangiomas via radical resection was time consuming, leading to a large amount of blood loss. Even after preoperative embolization, the intraoperative blood loss was as high as 2420 ml (range = 1580–3400 ml), and the average surgery duration was 608 min (range = 480–700 min). Goldson et al. [9] found that although hemangiomas were aggressive, their biological properties were benign, and they did not require en bloc resection to achieve a clear surgical margin. According to the current literature [24–27], pregnant patients are prone to hemangioma recurrence because of the significant elevation of vascular endothelial growth factor (VEGF) during pregnancy [28]. The invasiveness of hemangiomas may be related to VEGF, and the bone tissue of elderly patients has less VEGF [29]. Therefore, we recommend that en bloc resection surgery be not required for elderly patients.

Vertebral decompression drastically reduces intraoperative blood loss and technical requirements; however, it has a relatively high postoperative tumor recurrence rate. Acora et al. [2] examined 22 patients with S3 aggressive hemangiomas and found that 6 patients underwent simple decompression surgery, and two had recurrence rates of 33.3%. In a multicenter study, Goldson et al. [9] found that of 68 patients undergoing simple decompression surgery, three had tumor recurrence after surgery. Because the inclusion criterion of the Goldson et al. [9] study was symptomatic hemangioma (and not S3 aggressive hemangioma), it should not be directly compared with Acora et al. [3]. Additionally, this recurrence rate is acceptable because only three of 68 patients with symptomatic hemangiomas showed recurrence. In the reviewed literature (Table 4), we also found that only three elderly patients had tumor recurrence after decompression surgery. From the cases included in Acosta et al. [2] and Hekster et al. [18], we learned that the cause of recurrence was incomplete clearance of the intravertebral tumor due to the large amount of intraoperative blood loss where only the portion that compressed the spinal canal was removed. As early as 1995, Cotton et al. [35] reported the use of intraoperative bone cement to stabilize the spine. Wang et al. [14] showed that the use of bone cement to treat S3 aggressive hemangiomas significantly reduced intraoperative blood loss and postoperative recurrence. In that study, patients who received bone cement during the surgery had no recurrence. Moreover, with the recent rise of polymethyl methacrylate (PMMA) in patients undergoing decompression and resection, the intraoperative injection of bone cement might further reduce intraoperative blood loss [36]. However, some studies have also reported the risks of bone cement leakage [35, 37, 38]. In this study, we first proposed that the infusion of 1.0 mm × 1.0 mm × 1.0 mm absorbable gelatin sponge particles prior to the injection of bone cement might effectively reduce blood loss and provide a clearer visual field for surgery. Absorbable gelatin sponge embolization of venous channels before cement injection has not been widely used as a technique to prevent leakage. However, routine absorbable gelatin sponge embolization has been shown to be a safe and feasible method during vertebroplasty [39]. Absorbable gelatin sponge infusion prior to bone cement might effectively reduce the leakage of bone cement into the soft tissue, especially for patients with ruptured posterior vertebral body walls. Furthermore, absorbable gelatin sponge significantly reduces intraoperative blood loss and surgical duration compared with patients without absorbable gelatin sponge treatment.

**Table 4:** Summary of the management and recurrence of patients with vertebral hemangioma

Authors	No.	Age	Tumor types	Symptom	Tumor level	Treatment	FU (y)	Recurrence (y)	Blood loss (ml)	Risk factors of recurrence
Acosta et al. (2008) [2]	8	72	S3	Pain, Myelopathy	T5	TA	20	2.4		
	9	44	S3	Radiculopathy	T11、T12	decompression	12	11		
	11	66	S3	Radiculopathy	T8	Incomplete laminectomy	2.6	2.8		Excessive bleeding. Incomplete laminectomy
Akash et al. (2019) [30]	6	<60	SH	Pain	T	Radiotherapy	30	33.4		
Chandra et al. (2018) [31]	32	17	SH	Pain, Myelopathy, Radiculopathy	T7	Laminectomy, alcohol injection	2.5	2		
Cloran et al. (2015) [4]	1	23	S3	Pain, Radiculopathy	L5、S1–2	TA, decompression	1	1		
	15	73	S3	Pain, Radiculopathy	L1	Corpectomy	10	10		
Eichberg et al. (2017) [32]	7	52	S3	Paraparesis	T7	Decompression	3.6	1	2600	Excessive bleeding
Goldstein et al.(2015) [9]		39	SH	Pain	L5	Intralesional excision and adjuvant radiotherapy	3.9	4.4		
		50	SH	Pain	T6–T8	Intralesional excision without adjuvantradiotherapy	3.9	5.3		
		39	S3	Pain, Paraparesis	T6	TA, laminectomy and intralesional tumor debulking	13.5	5		
Hekster et al. (1972) [18]		61	S3	Paraparesis	T7	lamineetorny		3		Excessive bleeding
Jiang et al. (2014) [10]	12	24	S3	Myelopathy	T7	Spondylectomy	4.5	1.2		Excessive bleeding Without radiotherapy
	13	37	S3	Pain, Myelopathy	T10	Decompression	4.4	1		Without radiotherapy
	22	55	S3	Myelopathy	T4–6	laminectomy	10.8	9		Without radiotherapy
Kato et al. (2010) [12]	2	51	S3	Pain, Paraparesis	T4	Decompression	24	14	1580	
Mayank et al. (1999) [33]	5	35	SH	Paraparesis, Myelopathy	T7	Alcohol ablation	2.0	0.1		
Urrutia et al. (2011) [11]	1	14	S3	Radiculopathy	T12	Decompression	2.0	1.25		
Wang et al. (2017) [34]			S3			Decompression	5.3	5.4		
Wang et al. (2018) [14]		16	S3	Radiculopathy	T10	Incomplete vertebrectomy	4	0.4		

*Abbreviations*: *S3* Enneking stage 3, *SH* symptomatic vertebral haemangiomas, *TA* transarterial embolization, *FU* follow-up

Additionally, we believe that invasiveness was relatively poor among elderly patients with aggressive hemangiomas. Our literature review showed that the entirety of tumor clearance and the clarity of the intraoperative visual field were also factors affecting tumor recurrence. Therefore, we believe that decompression is sufficient for elderly patients, en bloc resection is not necessary to prevent tumor recurrence, and preoperative embolization should be used whenever possible in the clinic.

This single-center, retrospective study had a small sample size. Long-term prospective studies with larger samples are required for more detailed clinical staging and data evaluation of patients with aggressive vertebral hemangioma.

## Conclusions

Multimodal treatment significantly alleviates the clinical symptoms of elderly patients with aggressive hemangiomas. The intraoperative injection of absorbable gelatin sponge may be a safe and effective method to reduce blood loss and surgery duration. The recurrence of aggressive hemangioma might be associated with aging.

## Data Availability

All data generated or analyzed during this study are available upon reasonable request from the corresponding author.

## Abbreviations

BMI: body mass index
CT: computed tomography
Group A: decompression combined with intraoperative vertebroplasty
Group B: decompression combined with intraoperative vertebroplasty and absorbable gelatin sponge
MRI: magnetic resonance imaging.
S1: Enneking stage 1
S2: Enneking stage 2
S3: Enneking stage 3
VAS: visual analogue scale
